# Genome Announcement: Further Improved Genome Assembly of *Parapristionchus giblindavisi*

**DOI:** 10.2478/jofnem-2025-0026

**Published:** 2025-06-21

**Authors:** Waltraud Röseler, Kohta Yoshida, Christian Rödelsperger

**Affiliations:** Department for Integrative Evolutionary Biology, Max Planck Institute for Biology, Max-Planck-Ring 9, 72076 Tübingen, Germany; Current address: Department of System Pathology for Neurological Disorders, Brain Research Institute, Niigata University, Niigata, Japan

**Keywords:** Comparative genomics, Evolution, Transposons, Nigon elements, Pristionchus pacificus

## Abstract

Nematodes such as *Caenorhabditis elegans* and *Pristionchus pacificus* are powerful models for associating phenotypes to genotypes. However, exploring the evolution of identified genetic loci requires a robust phylogenomic framework. Here, we present an updated genome of the nematode *Parapristionchus giblindavisi* which is the only known member of the sister group of *Pristionchus*. Reassembly of previously generated long read sequencing data combined with new Hi-C data resulted in a near chromosome-scale genome assembly spanning 302.5Mb. The Hi-C contact map, karyotyping data and comparative genomic analysis support an organization of the *P. giblindavisi* genome into six chromosomes, whereby all autosomes correspond to individual Nigon elements and the sex chromosome represents a fusion of Nigon elements D and X. The further improved *P. giblindavisi* genome will be useful as an outgroup for dating and polarizing lineage-specific genomic signatures.

Almost 30 years ago, the free-living nematode *Pristionchus pacificus* was introduced as a satellite model organism for comparative studies with *Caenorhabditis elegans* (Sommer et al., 1996). It shares features with *C. elegans* like hermaphroditism, transparency, short generation time, and small genome size, which made it a similar powerful model system to identify the genetic basis of several traits and to make inference about the evolution of the underlying regulatory programs. Even evolutionary analyses at finer resolutions were possible due to continuous sampling efforts that led to a collection of more than 50 *Pristionchus* species and multiple new genera of diplogastrid nematodes (Herrmann et al., 2024; Kanzaki et al., 2021; Ragsdale et al., 2014; Herrmann et al., 2013). In order to establish a phylogenomic framework to explore the evolution of genes within this family of nematodes, we sequenced the genomes of several species (Prabh et al., 2018). One of those species was *Parapristionchus giblindavisi* that had originally been found on a beetle in Japan. *P. giblindavisi* shows a couple of morphological differences with regard to *Pristionchus* nematodes and phylogenetic and phylogenomic analyses revealed deep divergence from other genera (Kanzaki et al., 2012; Rödelsperger et al., 2024). Since the original genome assembly was highly fragmented, we recently resequenced the *P. giblindavisi* strain RS5555B using the Pacific Biosciences single molecule long read sequencing platform (Röseler et al., 2022). However, despite a 60X long read sequencing coverage with mean read length of 13kb, the resulting assembly was still fragmented into 735 contigs with an N50 value of 791kb. Here, we present a further improved *P. giblindavisi* genome based on reassembly and chromosome conformation capture. Specifically, we generated and sequenced a Hi-C library as described previously (Rödelsperger et al., 2024). This yielded over 52 million paired-end reads. We then combined this data with the previously generated long reads to generate a haplotype-resolved raw assembly with the software Hifiasm (version 0.16.1-r375 with -l3 option) (Cheng et al., 2021). This resulted in a primary haplotype of 307.1Mb (998 contigs with N50=890.0kb) and a secondary haplotype comprising 80.7Mb of allelic variation. We then aligned the Hi-C data to the primary haplotype with the help of the BWA mem program (version 0.7-17-r1188) (Li and Durbin, 2009). More than 95% of read pairs were mapped to the assembly, which allowed us to investigate the range distribution of chromatin interactions. While most interactions were identified in the range between 100bp and 1kb, we detected millions of read pairs spanning larger genomic distances up a megabase. This allowed us to scaffold the assembly with the yahs tool (version 1.2.a.2) (Zhou et al., 2023) followed by manual inspection and curation after visualizing the Hi-C contact map (Fig. 1A) and removal of a 4.6Mb scaffold representing the dietary bacteria *Escherichia coli* OP50. Only six of the 404 scaffolds were larger than 10Mb and accounted for 88.8% of the total assembly (303.1Mb). This translates into an N50 value of 43.6Mb (Table 1). These results suggest that *P. giblindavisi* has six chromosomes. This was confirmed by karyotypic analysis of male sperm and meiotic cells (Fig. 1B) using previously described methods (Yoshida et al., 2024). Among the unplaced contigs, we found one candidate (scaffold_145) that showed >85% nucleotide level sequence identity with the mitochondrial genome from *P. pacificus* (Genbank accession: NC_015245.1) (Molnar et al., 2011). Evidence-based gene annotations were generated for the unmasked assembly using the PPCAC pipeline (version 1) (Rödelsperger, 2021). This pipeline used previously generated RNA-seq data for *P. giblindavisi* (Prabh et al., 2018) as transcriptomic evidence and the community-curated gene annotations for *P. pacificus* (Athanasouli et al., 2020) as homology data, but it does not involve any gene prediction software. This resulted in 22,594 gene models of which 74% are derived from RNA-seq data. The BUSCO completeness value was estimated to be 88.4% (version 5, eukaryota_odb12) including 1.6% duplicates (Simão et al., 2015). We utilized the gene models to visualize the distribution of Nigon elements across the chromosomes (Fig. 1C). These Nigon elements correspond to seven ancestral linkage blocks that have been repeatedly recombined during the evolution of the nematode order Rhabditida yielding various chromosomal configurations (Tandonnet et al., 2019; Rödelsperger, 2024). All seven Nigon elements are largely intact in the *P. giblindavisi* genome, which supports the overall integrity of the assembled chromosomes. The fusion between Nigon elements X and D is consistent with the previously identified increased fusion rate of the sex-linked Nigon element X (Gonzalez de la Rosa et al., 2021). Finally, we investigated repetitive sequences in order to explore why the genome of *P. giblindavisi* is almost twice the size of *P. pacificus*, but has over 7000 genes less (Athanasouli et al., 2020). The software Red (version 05/22/2015) estimated the repeat content of the *P. giblindavisi* genome to be 56.1% as compared to 23.9% for the *P. pacificus* genome (Athanasouli and Rödelsperger, 2022; Girgis, 2015). Thus, the increased genome size of the *P. giblindavisi* can likely be explained by large-scale lineage-specific activity of transposons.

**Figure 1:**
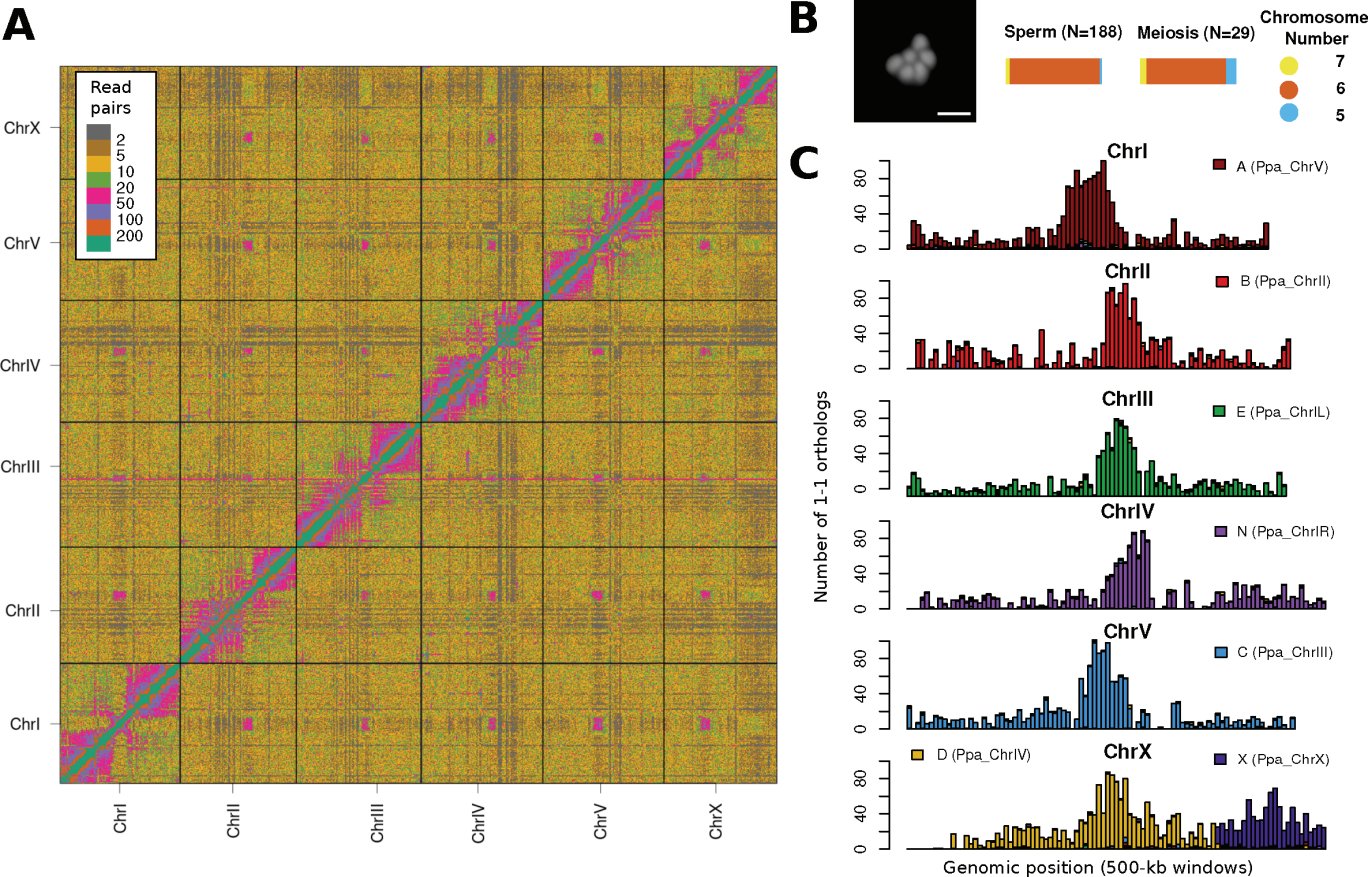
(A) The heatmap shows the Hi-C contact map for the *P. giblindavisi* genome. The color code for each cell indicates the number of Hi-C read pairs identified for a specific pair of 250-kb windows. (B) The picture shows a sperm cell of *P. giblindavisi* whereby the chromosomes were stained by Hoechst 33342. The scale bar indicates 2 μm. The bar plots show the proportion of male meiotic cells and sperms with each chromosome number. (C) We defined 1-1 orthologs between *P. giblindavisi* and *P. pacificus* proteins using best reciprocal BLAST hits and assigned Nigon elements based on the location of the 1-1 ortholog in the *P. pacificus* genome. Each bar shows the number of orthologs from different Nigon elements across 500-kb windows of the *P. giblindavisi* chromosomes.

**Table 1:** Characteristics of different *P. giblindavisi* genome assemblies.

	*P. giblindavisi* (Röseler et al., 2022)	*P. giblindavisi* (This study)
Number of contigs/scaffolds	735	404
Total genome size (Mb)	251.6	303.1
N50 (Mb)	0.8	43.6
Number of genes	22,488	22,594
BUSCO (proteins) (%)	87.6	88.4
Median protein length (amino acids)	224 [127–389]	222 [127–387]
Median exon number	7 [4–11]	6 [4–11]
Median exon length (nucleotides)	93 [71–120]	93 [71–120]
Median intron length (nucleotides)	132 [54–297]	132 [54–297]

BUSCO values include single copy and duplicated genes. The number in brackets after the median values denote the interquartile range.

The raw Hi-C sequencing data and the *P. giblindavisi* genome assembly have been uploaded to the European nucleotide archive under the study accession PRJEB87829. The data are also available on the pristionchus.org webserver.
